# Retraction: Mental health problems among front-line healthcare workers caring for COVID-19 patients in Vietnam: a mixed methods study

**DOI:** 10.3389/fpsyg.2024.1378832

**Published:** 2024-02-07

**Authors:** Frontiers Editorial Office

The journal retracts the 15th April 2022 article cited above.

Following concerns regarding the originality of the article, an investigation was conducted in accordance with Frontiers' policies. It was found that the complaint was valid and that the article should be retracted due to an unacceptable level of similarity to another article published by the same authors: Bui, T. T., Tran, T. L., Nguyen, K. T., Tran, T. N., Do, T. M., Pham, A. T., and Tran, T. T. H. (2021). Several factors are associated with depression among hospital staff directly caring for COVID-19 patients. Journal of Medical Research, 145(9), 69–76. https://doi.org/10.52852/tcncyh.v145i9.265.

This retraction was approved by the Chief Editors of Psychology and the Chief Executive Editor of Frontiers. The authors do not agree to this retraction.

